# Targeting EZH1 and EZH2 contributes to the suppression of fibrosis-associated genes by miR-214-3p in cardiac myofibroblasts

**DOI:** 10.18632/oncotarget.13048

**Published:** 2016-11-03

**Authors:** Wen-Si Zhu, Chun-Mei Tang, Zhen Xiao, Jie-Ning Zhu, Qiu-Xiong Lin, Yong-Heng Fu, Zhi-Qin Hu, Zhuo Zhang, Min Yang, Xi-Long Zheng, Shu-Lin Wu, Zhi-Xin Shan

**Affiliations:** ^1^ Guangdong Cardiovascular Institute, Guangdong Provincial Key Laboratory of Clinical Pharmacology, Guangzhou, China; ^2^ Guangdong General Hospital, Guangdong Academy of Medical Sciences, Guangzhou, China; ^3^ Southern Medical University, Guangzhou, China; ^4^ School of Medicine, South China University of Technology, Guangzhou, China; ^5^ The Libin Cardiovascular Institute of Alberta, Department of Biochemistry & Molecular Biology, The University of Calgary, Calgary, Canada

**Keywords:** microRNA-214-3p, cardiac fibrosis, cardiac myofibroblast, EZH1, EZH2, Pathology Section

## Abstract

The role of microRNA-214-3p (miR-214-3p) in cardiac fibrosis was not well illustrated. The present study aimed to investigate the expression and potential target of miR-214-3p in angiotensin II (Ang-II)-induced cardiac fibrosis. MiR-214-3p was markedly decreased in the fibrotic myocardium of a mouse Ang-II infusion model, but was upregulated in Ang-II-treated mouse myofibroblasts. Cardiac fibrosis was shown attenuated in Ang-II-infused mice received tail vein injection of miR-214-3p agomir. Consistently, miR-214-3p inhibited the expression of Col1a1 and Col3a1 in mouse myofibroblasts *in vitro*. MiR-214-3p could bind the 3′-UTRs of enhancer of zeste homolog 1 (EZH1) and −2, and suppressed EZH1 and −2 expressions at the transcriptional level. Functionally, miR-214-3p mimic, in parallel to EZH1 siRNA and EZH2 siRNA, could enhance peroxisome proliferator-activated receptor-γ (PPAR-γ) expression and inhibited the expression of Col1a1 and Col3a1 in myofibroblasts. In addition, enforced expression of EZH1 and −2, and knockdown of PPAR-γ resulted in the increase of Col1a1 and Col3a1 in myofibroblasts. Moreover, the NF-κB signal pathway was verified to mediate Ang-II-induced miR-214-3p expression in myofibroblasts. Taken together, our results revealed that EZH1 and −2 were novel targets of miR-214-3p, and miR-214-3p might be one potential miRNA for the prevention of cardiac fibrosis.

## INTRODUCTION

Cardiac fibrosis participates in many cardiac pathophysiologic processes, with the characterizations of proliferation of cardiac fibroblasts and excessive accumulation of extracellular matrix in myocardium [1]. The initial reparative fibrosis is crucial for preventing rupture of the ventricular wall, however, the exaggerated fibrotic response contributes to progressive impairment of cardiac function, heart failure, fatal arrhythmia and sudden cardiac arrest [2]. Cardiac fibroblasts are the most prevalent cell type in the heart, upon injury and the stimulations of fibrogenic mediators, these cells transform to more active myofibroblast phenotype. Myofibroblasts are the main effector cells in cardiac fibrosis, which express contractile proteins and exhibit migratory, proliferative and secretory properties [3]. Currently, no efficient therapeutic approach is available for inhibiting cardiac fibrosis. Understanding the mechanisms responsible for cardiac fibrosis is crucial to set up anti-fibrotic therapy strategies for patients with heart diseases.

Enhancer of zeste homolog 2 (EZH2) is the enzymatic subunit of polycomb repressive complex 2 (PRC2), a complex that methylates lysine 27 of histone H3 (H3K27) to promote chromatin compaction and gene silencing [4, 5]. EZH1, a homolog of EZH2, catalyzes addition of methyl groups on H3K27 and prevents the derepression of PRC2 target genes. EZH1 plays a complementary but nonredundant roles for EZH2 in mediating H3K27 methylation and gene repression [6]. EZH2 was shown upregulated in hepatic fibrosis, along with the increase of H3K27me3 in the promoter of PPAR-γ and downregulation of PPAR-γ [7]. Specifically, PPAR-γ inhibits the expression of α-SMA, type I collagen, and TGF-β1 in hepatic stellate cells [8]. PPAR-γ agonists were proved to inhibit TGF-β signal transduction and were potential antifibrogenic agents in many organs including the liver, lung, kidney, skin and heart [9, 10]. However, the exact roles of EZH1, -2 and PPAR-γ in cardiac fibrosis are still unclear.

MicroRNAs (miRNAs) are endogenous, non-coding, 20-23 nucleotide RNAs that negatively regulate a variety of target genes involved in cardiovascular physiology and diseases [11–13]. Recent studies have demonstrated that miRNAs are implicated in myocardial fibrosis; for example, miR-21, -29, -30, -133, -433 and -590 modulate fibrosis-related genes expression in animal models of ischemia/reperfusion myocardial infarction, trans-aortic constriction(TAC), nicotine-induced atrial fibrosis, respectively [14, 15]. MicroRNA-214 (miR-214) was shown involved in the pathogenesis of cardiac fibrosis [16–19], but the role of miR-214 in cardiac fibrosis has not yet been well understood.

In the present study, we investigated the effect and potential targets of miR-214-3p in Ang-II-induced fibrosis *in vivo* and *in vitro*. Our data demonstrated the anti-fibrotic effect of miR-214-3p by targeting EZH1 and -2, resulting in increase of PPAR-γ and suppression of Col1a1 and Col3a1 in mouse myofibroblasts. The NF-κB signal pathway was verified to mediate the upregulation of miR-214-3p in myofibroblasts exposed to Ang-II.

## RESULTS

### Decreased expression of miR-214-3p in the fibrotic mouse myocardium

**Figure 1:**
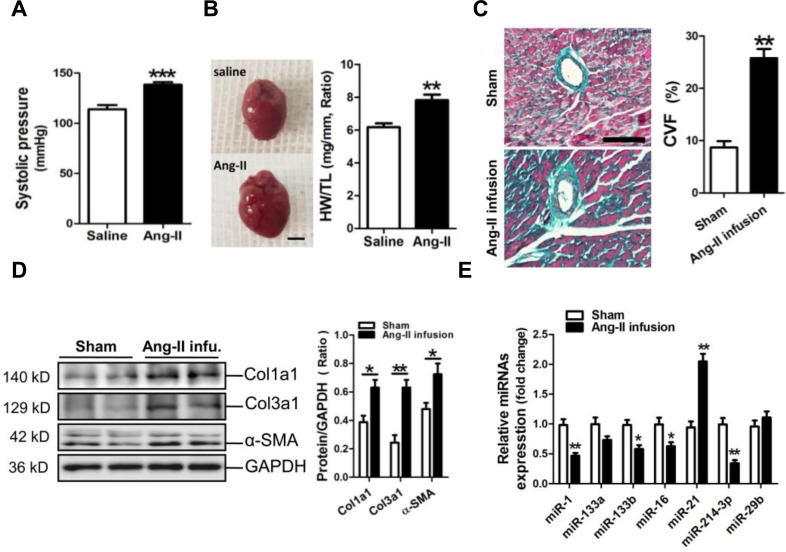
MicroRNA-214-3p (miR-214-3p) expression in the fibrotic myocardium of a mouse Ang-II infusion model **A.** The systolic pressure measurement. **B.** The ratio of heart weight/tibia length (HW/TL). The scale bar was 2 cm.**C.** Masson trichrome stainning. The scale bar was 100 μm. **D.** Expressions of Col1a1, Col3a1 and α-SMA in mouse myocardium by Western blot assay. **E.** Expressions of mir-1, -133a, -133b, 16, -21, -214-3p and -29b in mouse myocardium by RT-qPCR assay. Data are shown as mean ± sem, *p < 0.05, **p < 0.01, ***p < 0.001. N = 5-8.

An animal model of myocardial fibrosis was established in mice received Ang-II infusion for two weeks. The HW/TL data indicated that a mouse model of cardiac remodeling was well established, with the increases in the systolic pressure and in the ratio of HW/TL (*p* < 0.001, *p* < 0.01, respectively) (Figure 1A, 1B). Masson staining results revealed that the perivascular fibrosis was significantly increased in the myocardium of a mouse Ang-II infusion model (*p* < 0.01) (Figure 1C). Consistently, results of Western blot assay showed that Col1a1, Col3a1 and α-SMA were markedly increased in the fibrotic mouse myocardium (*p* < 0.05, *p* < 0.01, respectively) (Figure 1D). We determined the concerned miRNAs, including miR-1, -133a, -133b, -21, -214-3p and -29b, in the fibrotic mouse myocardium. RT-qPCR results indicated that miR-21 was upregulated, but miR-1, -133b, -16 and -214-3p were downregulated in Ang-II-induced mouse myocardium (*p* < 0.05, *p* < 0.01, respectively) (Figure 1E).

### MiR-214-3p attenuates Ang-II-induced fibrotic phenotype *in vivo*

**Figure 2:**
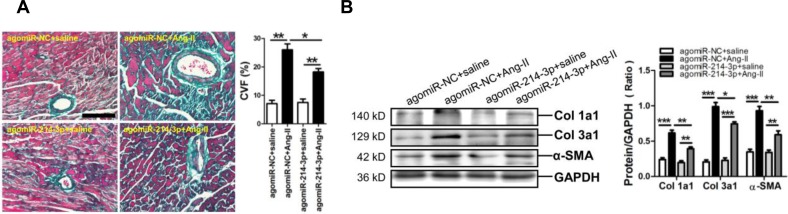
Phenotype of myocardial fibrosis of a mouse Ang-II infusion model with enforced expression of miR-214-3p **A.** Representative sections of mouse myocardium with Masson trichrome staining. The scale bar is 100 μm. **B.** Expressions of Col1a1, Col3a1 and α-SMA in mouse myocardium by Western blot assay. Data are shown as mean ± sem, *p < 0.05, **p < 0.01, ***p < 0.001. N = 5-8.

To further demonstrate the potential role of miR-214-3p in Ang-II-induced myocardial fibrosis, we determined whether restoring miR-214-3p expression *via* tail vein injection of miR-214-3p agomir could exert protective effect on the myocardial fibrosis. As expected, the level of miR-214-3p was significantly increased in the myocardium of mice received injection of miR-214-3p agomir (Supplimentary Figure 1). The Masson staining results revealed that myocardial fibrosis was markedly increased in Ang-II infusion mice, but which could be reversed by enforced expression of miR-214-3p (*p* < 0.05, *p* < 0.01, respectively) (Figure 2A). Meanwhile, our western blot results demonstrated that expression of Col1a1, Col3a1 and α-SMA in mouse myocardium in response to Ang-II infusion was also suppressed by miR-214-3p injection (*p* < 0.05, *p* < 0.01, respectively) (Figure 2B).

### MiR-214-3p attenuates expression of fibrosis-related genes in myofibroblasts

**Figure 3:**
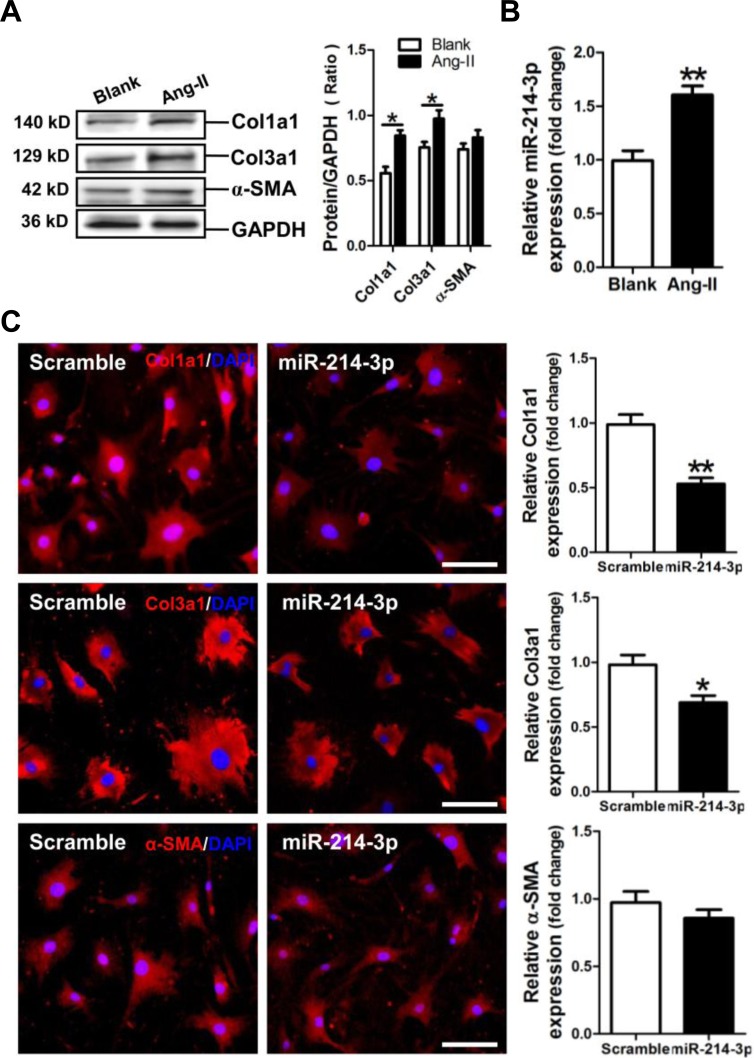
Expression of miR-214-3p in Ang-II-induced myofibroblasts and its effect on expressions of Col1a1, Col3a1 and α-SMA in myofibroblasts **A.** Expressions of Col1a1, Col3a1 and α-SMA in Ang-II-induced myofibroblasts by Western blot assay. **B.** Expression of miR-214-3p in Ang-II-induced myofibroblasts by RT-qPCR assay. **C.** Expressions of Col1a1, Col3a1 and α-SMA in miR-214-3p-modified myofibroblasts by FIHC assay. The scale bar is 100 μm. Data are shown as mean ± sem, *p < 0.05, **p < 0.01. N = 3.

We established a cell model of Ang-II-induced myofibroblast fibrosis, resulting in significant increases of Col1a1 and Col3a1 protein expression (*p* < 0.05) (Figure 3A). Meanwhile, miR-214-3p was observed upregulated in Ang-II-induced myofibroblasts (*p* < 0.01) (Figure 3B). Results of FIHC assay revealed that Col1a1 and Col3a1 expression was significantly suppressed in miR-214-3p-modified mouse myofibroblasts (*p* < 0.05, *p* < 0.01, respectively) (Figure 3C).

### Verification of EZH1 and -2 as target genes of miR-214-3p

**Figure 4:**
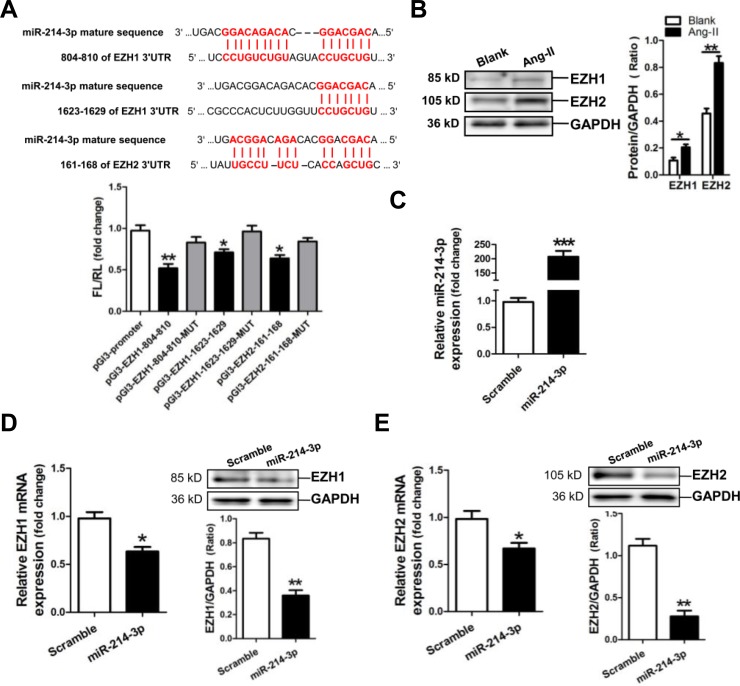
MicroRNA-214-3p (miR-214-3p) negatively modulates EZH1 and -2 expression **A.** Verification of EZH1 and -2 as a target gene of miR-214-3p by the dual luciferase reporter system. Predicted miR-214-3p seed matches to the sequence in the 3′UTR of EZH1 and -2 mRNA. The seed sequence of miR-214-3p is CAGCAGG, and the complementary nucleotide sequences are shown in red words. Data are shown as mean ± sem, *p < 0.05, **p < 0.01 *vs* pGl3-promoter vector control, N = 3. **B.** EZH1 and -2 expression in Ang-II-induced myofibroblasts by Western blot assay. **C.** Determination of miR-214-3p level in miR-214-3p-modified myofibroblasts by RT-qPCR assay. MRNA and protein expressions of EZH1 **D.** and EZH2 **E.** in miR-214-3p-modified myofibroblasts by RT-qPCR and Western blot assay, respectively. Data are shown as mean ± sem, *p < 0.05, **p < 0.01, ***p < 0.001. N = 3.

Analysis of the databases Mirdb (www.mirdb.org) and TargetScan-Vert (www.targetscan.org) showed that EZH1 and -2 were potential target genes of miR-214-3p. The matching positions for miR-214-3p within 3′-UTR of the targeted mRNAs are shown in Figure 4A. The dual luciferase assay demonstrated that miR-214-3p significantly reduced the luciferase activities through binding the following sites, including 804-810, 1623-1629 of EZH1 3′-UTR, 161-168 of EZH2 3′-UTR (*p* < 0.05, *p* < 0.01, respectively) (Figure 4A).

Next, we detected the expressions of EZH1 and -2 in Ang-II-induced mouse myofibroblasts. Western blot result revealed that the expressions of EZH1 and -2 were markedly increased in Ang-II-induced mouse myofibroblasts (*p* < 0.05, *p* < 0.01, respectively) (Figure 4B). Then, we examined the expression of EZH1 and -2 in mouse myofibroblasts transfected with miR-214-3p mimic. RT-qPCR result showed that the level of miR-214-3p was dramatically increased in miR-214-3p-modified myofibroblasts (*p* < 0.001) (Figure 4C). Compared with the negative scramble control, mRNA and protein expression of EZH1 and -2 were significantly decreased in miR-214-3p-modified myofibroblasts (*p* < 0.05, *p* < 0.01, respectively) (Figure 4D, 4E).

### MiR-214-3p, EZH1 siRNA and EZH2 siRNA attenuate expressions of Col1a1 and Col3a1 in myofibroblasts

**Figure 5:**
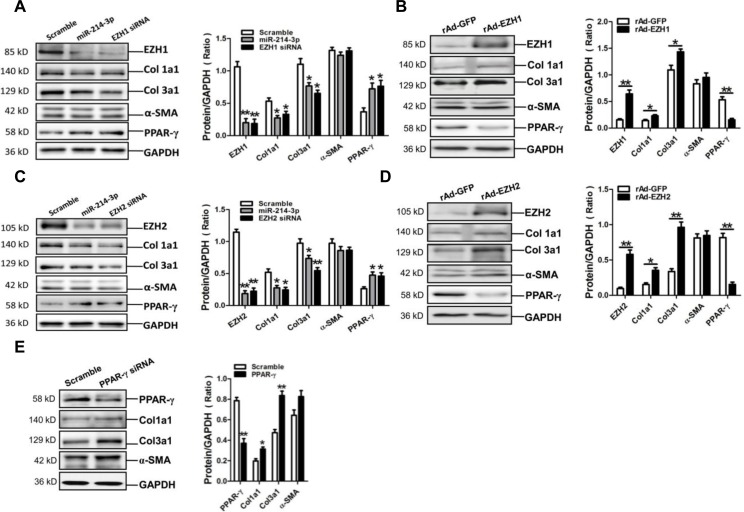
Expressions of Col1a1, Col3a1 and α-SMA in mouse myofibroblasts with transfection of miR-214-3p mimic, EZH1 siRNA, EZH2 siRNA and PPAR-γ siRNA, or overexpression of EZH1 and -2, respectively **A.** Protein expression of EZH1, Col1a1, Col3a1, α-SMA and PPAR-γ in myofibroblasts with transfection of miR-214-3p mimic, EZH1 siRNA, respectively. **B.** Expression of EZH1, Col1a1, Col3a1, α-SMA and PPAR-γ in myofibroblasts with enforced expression of EZH1 by Western blot assay. **C.** Protein expression of EZH2, Col1a1, Col3a1, α-SMA and PPAR-γ in myofibroblasts with transfection of miR-214-3p mimic, EZH2 siRNA, respectively. **D.** Expression of EZH2, Col1a1, Col3a1, α-SMA and PPAR-γ in myofibroblasts with enforced expression of EZH2 by Western blot assay. **E.** Protein expression of PPAR-γ, Col1a1, Col3a1 and α-SMA in myofibroblasts with transfection of PPAR-γ siRNA. Data are shown as mean ± sem, *p < 0.05, **p < 0.01 *vs* scramble control, N = 3 in **A.**, **C.**, **E.** *p < 0.05, **p < 0.01 *vs* rAd-GFP control, N = 3 in **B.**, **D.**

MiR-214-3p mimic and EZH1 siRNA were transfected into mouse myofibroblasts. Results of western blot assay showed that miR-214-3p mimic and EZH1 siRNA could efficiently suppress the expressions of Col1a1 and Col3a1, and increase the expression of PPAR-γ, without significant effect on the expression of α-SMA in myofibroblasts (*p* < 0.05, *p* < 0.01, respectively) (Figure 5A). Moreover, enforced expression of EZH1 could markedly enhance the expressions of Col1a1 and Col3a1, but suppress the expression of PPAR-γ in myofibroblasts (*p* < 0.05, *p* < 0.01, respectively) (Figure 5B).

Then, miR-214-3p mimic and EZH2 siRNA were also transfected into mouse myofibroblasts. Western blot assay showed that miR-214-3p mimic and EZH2 siRNA could markedly suppress the expressions of Col1a1 and Col3a1, and increase the expression of PPAR-γ, without significant effect on the expression of α-SMA in myofibroblasts (*p* < 0.05, *p* < 0.01, respectively) (Figure 5C). In addition, overexpression of EZH1 could enhance the expressions of Col1a1 and Col3a1, but suppress the expression of PPAR-γ in myofibroblasts (*p* < 0.05, *p* < 0.01, respectively) (Figure 5D).

Furthermore, PPAR-γ siRNA was transfected into mouse myofibroblasts. Results of western blot assay demonstrated that PPAR-γ siRNA could significantly increase the expressions of Col1a1 and Col3a1, without significant effect on the expression of α-SMA in myofibroblasts (*p* < 0.05, *p* < 0.01, respectively) (Figure 5E).

### MiR-214-3p is up-regulated by Ang-II through the NF-κB pathway in myofibroblasts

**Figure 6:**
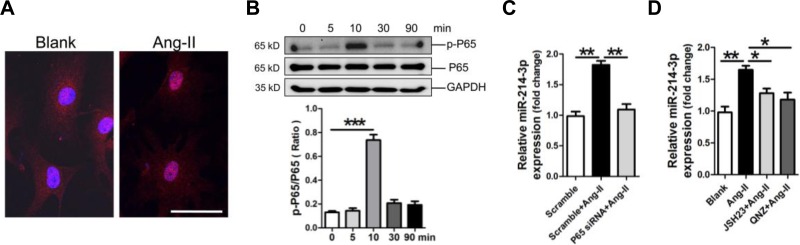
Up-regulation of microRNA-214-3p (miR-214-3p) in mouse myofibroblasts through NF-kB pathway **A.** Location of NF-κB P65 by FIHC assay. The scale bar is 50 μm. **B.** Activation of NF-kB signaling in Ang-II-treated myofibroblasts in a time-course study. MiR-214-3p expression in Ang-II-induced myofibroblasts with knockdown of P65 **C.**, or with pre-treatment with NF-kB inhibitor JSH23 and QNZ, respectively **D.**, was assessed by RT-qPCR assay. Data are shown as mean α sem, *p < 0.05, **p < 0.01, ***p < 0.001. N = 3.

Previous report showed that NF-κB activation suppresses miR-214 transcription in hepatocellular carcinoma cells [19]. In the current study, we investigated whether modulation of miR-214-3p in myofibroblats was also mediated through the NF-κB pathway. The FIHC result revealed the translocation of NF-κB P65 into nuclear of myofibroblats post-treatment with Ang-II (Figure 6A). Consistently, the result of western blot showed that the phosphorylation level of NF-κB P65 was significantly increased at 10 min in myofibroblats in response to Ang-II treatment (Figure 6B). Knockdown of P65 by P65 siRNA inhibited Ang-II-promoted miR-214-3p expression in myofibroblats (Figure 6C). Next, we pre-treated myofibroblats with NF-κB P65 inhibitor JSH23 or QNZ for 0.5 h before 3-hour Ang-II treatment. The RT-qPCR result demonstrated that treatment with either JSH23 or QNZ prevented Ang-II-induced miR-214-3p expression (Figure 6D). Collectively, our data suggest that up-regulation of miR-214-3p in Ang-II-induced myofibroblats results from the activation of NF-κB signaling.

## DISCUSSION

In the present study, miR-1, -133b and -16 were decreased, but miR-21 was increased in the fibrotic myocardium of a mouse Ang-II infusion model, which were consistent with previous reports [20–25]. Contrast to the previous reports that miR-214 was upregulated in the myocardium of a rat AAC model, and of a mouse ischemia/reperfusion (IR) injury model and of a rat isoproterenol injection model [16–19], our present data showed that miR-214-3p was significantly down-regulated in the myocardium of a mouse Ang-II infusion model, and was upregulated in Ang-II-induced mouse myofibroblasts. This opposite results of miR-214 expression in the fibrotic myocardium *in vivo* may result from different types of animal models.

Absence of miR-214 was shown to cause loss of cardiac contractility and increase of apoptosis in response to IR injury in mice. Mechanistically, the cardioprotective role of miR-214 was attributed to its repression on sodium/calcium exchanger 1 (Ncx1) and regulation of cardiomyocyte Ca^2^+ homeostasis [16]. And the anti-fibrotic effect of miR-214 was also reported in a rat model of acute myocardial infarction (AMI) [19]. However, one study showed that downregulation of miR-214 by antagonists attenuated cardiac fibroblast proliferation and collagen synthesis *via* inhibition of Mfn2 and activation of ERK1/2 MAPK signaling [18]. Similarly, knockdown of miR-214 *in vivo* using a specific antagomir (miR-214 inhibitor) prevented cardiac remodeling and dysfunction in a mouse heart failure model of pressure overload [26]. Collectedly, the role and potential target gene of miR-214 in cardiac fibrosis have not been well-illustrated.

Since our data showed that miR-214-3p was markedly down-regulated in the myocardium of a mouse Ang-II infusion model. Thereafter, we delivered miR-214-3p agomir *via* tail vein to increase miR-214-3p level and to investigate the role of miR-214-3p in Ang-II-induced cardiac fibrosis. As expected, the increase of cardiac fibrosis could be reversed by miR-2014-3p *in vivo*. Consistently, miR-214-3p could efficiently inhibit expressions of Col1a1 and Col3a1 in mouse myofibroblasts. Therefore, our data have demonstrated the anti-fibrotic effect of miR-214-3p, instead of a pro-fibrotic effect, in cardiac fibrosis. This conclusion has been supported by previous studies showing the protective effect of miR-214 by inhibition of fibrosis [16, 19, 27].

EZH1 and -2, as the sole histone methyltransferase, methylates H3K27 and mediates transcriptional silencing [4, 5]. EZH1 was known to play a complementary but nonredundant role for EZH2 in mediating H3K27 methylation and gene repression [6]. EZH2 was shown to play an important role in the development of renal fibrosis by downregulating expression of Smad7 and PTEN to activate profibrotic signaling pathways [28]. In addition, EZH2 was also reported involved in hepatic fibrosis by suppressing PPAR-γ expression [7]. Notably, activation of PPAR-γ effectively inhibited cardiac remodeling process by suppressing Brg1 and TGF-β1 through NF-κB pathway [29]. Moreover, overexpression of PPAR-γ inhibited the expression of α-SMA, type I collagen, and TGF-β1 in hepatic stellate cells [8]. Accumulating evidence demonstrates that PPAR-γ agonists have potential to reduce fibrosis in many organs, including the liver, lung, kidney, skin and heart [9, 10]. However, the exact roles of EZH1, -2 and PPAR-γ in cardiac fibrosis remain unexplored.

We observed that EZH1 and -2 were significantly increased in Ang-II-treated mouse myofibroblasts. Knockdown of EZH1 and -2 suppressed expressions of Col1a1 and Col3a1, along with the increase of PPAR-γ in mouse myofibroblasts. Consistently, enforced expression of EZH1 or -2 increase Col1a1 and Col3a1 expression, which correlated with the decrease of PPAR-γ. The above data has been supported by the previous study showing that EZH1 played a complementary but nonredundant role for EZH2 in gene repression [6]. As expected, knockdown of PPAR-γ resulted in enhanced expressions of Col1a1 and Col3a1 in myofibroblasts, which was consistent with the recent report that inhibition of PPAR-γ enhanced AMI-induced cardiac fibrosis [30]. Our present data reveals that EZH1/EZH2-PPAR-γ pathway participates in modulation of extracellular matrix genes, including Col1a1 and Col3a1, in cardiac fibrosis.

The present study has provided several lines of evidence to support the notion that miR-214-3p inhibits expressions of fibrosis-related genes through targeting EZH1 and -2. First, the *in silico* prediction indicated that EZH1 and -2 were potential targets of miR-214-3p, and the dual luciferase assay revealed that miR-214-3p specifically bound to the 804-810, 1623-1629 sites in the 3′-UTR of EZH1, and the 161-168 site in the 3′-UTR of EZH2. Additionally, miR-214-3p mimic inhibited expressions of fibrosis-related genes through targeting EZH1 and -2 expressions at both mRNA and protein levels in myofibroblasts. Moreover, in parallel with the findings with EZH1 siRNA and EZH2 siRNA, over-expression of miR-214-3p reduced the expression of Col1a1 and Col3a1, but enhanced the expression of PPAR-γ in myofibroblasts. Our present data has been supported by previous studies showing that EZH2 was a target of miR-214-3p [31, 32]. Notably, we further revealed EZH1, as another important target of miR-214-3p, mediated the anti-fibrotic effect of miR-214-3p by suppressing PPAR-γ expression in mouse myofibroblasts.

In the present study, injection of miR-214-3p was shown to inhibit Ang-II-induced expressions of fibrosis-related genes, including Col1a1, Col3a1 and α-SMA, in mouse myocardium *in vivo*. However, the results of cellular experiments *in vitro* indicated that α-SMA, except for Col1a1 and Col3a1, was not dramatically up-regulated by enforced expression of EZH1, -2 and PPAR-γ siRNA, and was not markedly suppressed by miR-214-3p mimic, EZH1 siRNA, EZH2 siRNA, as well. We also observed that expression of α-SMA couldn't be efficiently promoted in the third passage of mouse myofibroblasts, even post-Ang-II treatment. Compared with Col1a1 and Col3a1, the transcription activity of α-SMA was weak in the third passage of mouse myofibroblasts post-stimuli. Therefore, further investigations are needed to confirm and explore the mechanism underlying the low transcription activity of α-SMA in mouse myofibroblasts.

NF-κB signaling has been shown to play a role in cardiac remodeling [33–35]. Our present data has confirmed that the pathway involving NF-κB P65 was activated in Ang-II-treated mouse myofibroblasts. We used NF-κB P65 siRNA, NF-κB P65 inhibitor JSH23 and QNZ to further verify the participation of NF-κB P65 pathway in Ang-II-promoted upregulation of miR-214-3p in myofibroblasts. However, it was reported that miR-214 expression was negatively modulated by the NF-κB P65 pathway in hepatocellular carcinoma (HCC) cells [36]. This opposite finding may result from different types of cells. Nevertheless, the mechanism underlying the down-regulation of miR-214-3p in mouse fibrotic myocardium warrants further investigation.

**Figure 7:**
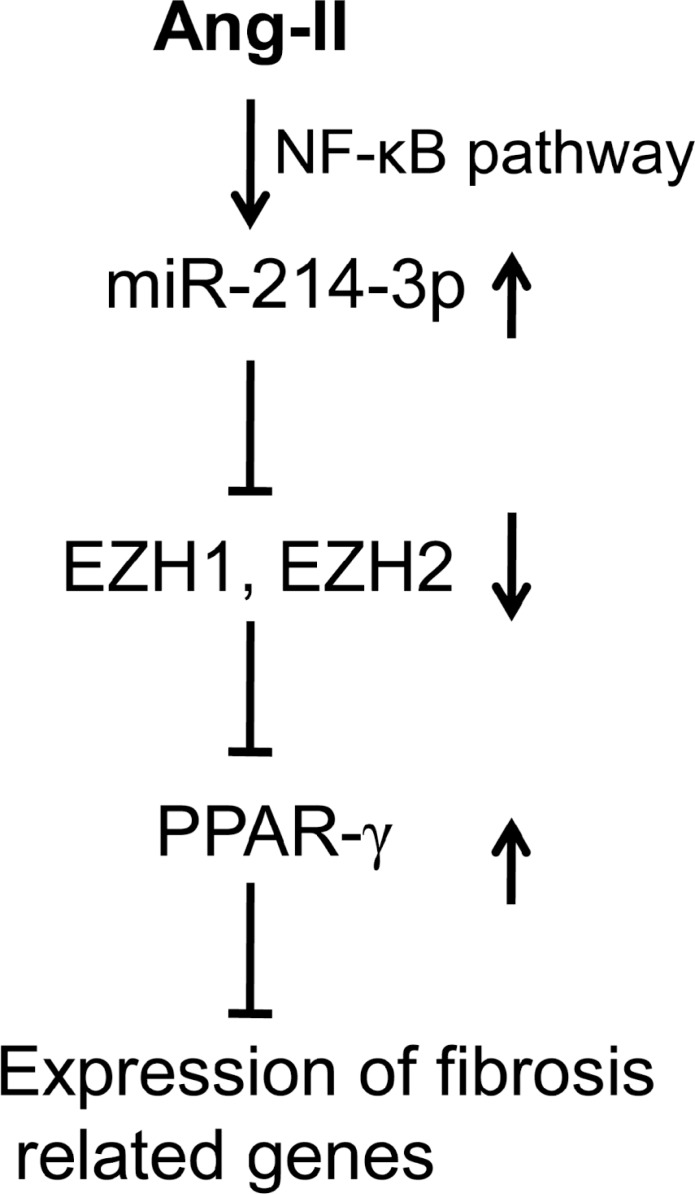
Schematic diagram of the mechanism whereby miR-214-3p exerts the anti-fibrotic effect in cardiac fibrosis MiR-214-3p is upregulated in Ang-II-induced myofibroblasts via NF-κB pathway. MiR-214-3p enhances PPAR-γ expression by targeting EZH1 and -2, resulting in the suppressing the expression of fibrosis related genes, including Col1a1 and Col3a1, in myofibroblasts.

Taken together, our results have demonstrated that miR-214-3p is down-regulated in cardiac fibrosis, and miR-214-3p ameliorates cardiac fibrotic responses *in vivo* and *in vitro*. Our data have also revealed that miR-214-3p inhibits fibrotic phenotype in cardiac myofibroblasts through down-regulation of EZH1 and -2. We also concluded that activation of the NF-κB signaling pathway contributes to the upregulation of miR-214-3p in Ang-II-induced myofibroblasts. Therefore, the present study suggests that miR-214-3p might be a potential target for prevention and treatment of cardiac fibrosis (as shown in Figure 7).

## MATERIALS AND METHODS

### Ethics statement

Male C57BL/6 mice weighing 20±3 g and 1- to 3-d old newborn C57BL/6 mice (License number SCXK (YUE) 2004-0011, Department of Experimental Animal Research Center, Sun Yat-sen University, Guangzhou, China) were used in the current studies. Mice were housed under a 12-h light/dark cycle under pathogen-free conditions and with free access to standard mouse chow and tap water. This study conformed to the Guide for the Care and Use of Laboratory Animals published by the US National Institutes of Health (8th Edition, National Research Council, 2011). The present program was also approved by the research ethics committee of Guangdong General Hospital (the approval number: No. GDREC2010093A).

### Animal studies

According to previously described methods, we established a mouse cardiac hypertrophy model of Ang-II (1.46 mg/kg/d, 14 d) infusion [37]. Mice were anesthetized through the intraperitoneal application of sodium pentobarbital (50 mg/kg), followed by implantation of the Ang-II mini-osmotic pump (alzet model 2002, Cupertino, CA, USA). The adequacy of anesthesia was confirmed by the absence of reflex response to foot squeeze. Body temperature was maintained at 37±0.5°C during surgery. At the end of the experiments, mice were killed with the intraperitoneal injection of an overdose of sodium pentobarbital (200 mg/kg).

To investigate the effect of miR-214-3p on Ang-II-induced fibrosis *in vivo*, 24 C57BL/6 mice were randomized into 4 groups: 1) agomiR-negative control (NC)+saline, 2) agomiR-NC+Ang-II (NC agomir with Ang-II infusion), 3) agomiR-214+saline (miR-214-3p agomir with saline infusion), and4) agomiR-214+Ang-II (miR-214-3p agomir with Ang-II infusion). All agomirs were purchased from (Guangzhou RiboBio, Guangzhou, China). The amount of 20 nmol NC agomir or 20 nmol miR-214-3p agomir was delivered into each mouse *via* tail vein injection at 4 interval time points within 14 d.

### Histological analysis

Mice were sacrificed with an overdose of sodium pentobarbital (200 mg/kg, ip) at the end of experiments. The mouse heart was excised, and the LV myocardium was fixed overnight in 10 % formalin. Samples were embedded in paraffin and cut into 4 μm thick sections. They were mounted on normal glass slides and stained with Masson trichrome for histological examination. For the collagen volume fraction (CVF) analysis, eight separate views (magnification = original×400) were selected and assessment of CVF used the following formula: CVF = collagen area/total area.

### Culture of mouse myofibroblasts and treatments

Mouse cardiac fibroblasts (CFs) were isolated from 1-3 days old C57BL/6 mice by using a modification of previous report [38]. Briefly, CFs were separated from cardiomyocytes by gravity separation and grown to confluency on 10-cm cell culture dishes in growth media (DMEM/LG 10% FBS, 1% penicillin and 1% streptomycin) at 37°C in humid air with 5% CO2. Myofibroblasts were achieved from CFs with the third passage and were used for experiments.

Myofibroblasts were incubated with 2×10^−5^ M Ang-II for 24 h to induce the fibrotic phenotype. Cells were treated with NF-κB inhibitor JSH23 (5 μM) or QNZ (5 nM). Cells were transfected with 50 nM scramble or miR-214-3p mimic, or 50 nM siRNA for EZH1, -2, PPAR-γ or NF-κB P65 (Ribobio, Guangzhou, China) by oligofectamine reagent (Invitrogen, Carlsbad, CA). As indicated, NMVCs were infected with the following recombinant adenovirus, respectively: rAd-GFP, rAd-EZH1 and rAd-EZH2 adenovirus (MOI 10).

### Fluorescence immunohistochemistry (FIHC)

Cultured myofibroblasts were washed in phosphate-buffered saline, fixed for 10 min in 3.7% formaldehyde, and permeabilized for 10 min in 0.1% Triton X-100. Cell monolayers were then washed in blocking solution and incubated with anti-Col1a1 antibody, anti-Col3a1 antibody, anti-α-SMA antibody (Santa Cruz Biotechnology, Santa Cruz, CA, USA), anti-NF-κB P65 (Cell SignalingTechnology, Beverly, MA, USA) over night at 4°C, respectively, followed by incubation with Alexa Fluor^®^ 555 donkey anti-rabbit IgG antibody (Molecular Probes, Eugene, OR, USA) for 1 h at room temperature. Confocal micrographs were obtained using a Leica SP5 confocal microscope (Leica, Mannheim, Germany). The fluorescence intensity analysis was performed using the LAS AFTCS SP5 imaging software.

### Quantitative miRNA and mRNA measurements

Quantitative reverse-transcription PCR (qRT-PCR) for miR-1, -133a,-133b,-16, -21, -214-3p and -29b was performed on cDNA generated from 0.5 μg total RNA according to the manufacturer's protocol (Ribobio, China). For the detection of mRNA expression of coding genes, the first-strand cDNAs were generated from 2.0 μg total RNA using a mixture of oligo (dT)_15_ and random primers with superscriptreverse transcriptase (Invitrogen, Carlsbad, CA). To normalize RNA content, U6 was used for miRNAs template normalization and GAPDH was used for coding genes template normalization. PCR was performed with the ViiA7 Quantitative PCR System (Applied Biosystems, Carlsbad, CA). The 2^−ΔΔCt^ method was used to calculate relative expression levels of the concerned miRNAs and coding genes [39]. PCR primers for miRNAs, U6 and coding genes are shown in Supplementary Table 1.

### Western blot analysis

The amount of 40-50 μg protein prepared from mouse myocardium or NMVCs was usedin a standard western blot analysis. The polyvinylidene fluoride (PVDF) membrane binding sample protein was incubated with a high affinity anti-Col1a1 antibody (1:1000), anti-Col3a1 antibody (1:1000), anti-α-SMA (1:2000) (Santa Cruz Biotechnology, USA), anti-EZH1 (1:1000), anti-EZH2 (1:1000), anti-PPAR-γ (1:1000) (Proteintech, Rosemont, IL, USA), anti-p-NF-κB P65 (1:1000), anti-NF-κB P65 (1:1000)(Cell SignalingTechnology, USA), respectively. An anti-GAPDH antibody (1:2000) (Santa CruzBiotechnology, USA) was used to detect the level of GAPDH as an internal control. Protein was visualized using the ECL Plus detection system (GE Healthcare, Waukesha, WI).

### Dual luciferase assay for EZH1 and -2 targets identification

According to our previous report [20], the recombinant luciferase reporter plasmid containing the potential miR-214-3p binding site sequences of EZH1 and -2 genes were constructed. Using a site-directed mutagenesis kit (TransGen, Beijing, China), the miR-214-3p binding site sequence CCUGCUG was replaced with CCACGAG, CCAGCUG was replaced with CCACGAG to construct the corresponding recombinant luciferase reporter plasmids containing the mutant potential miR-214 binding sequences. Human embryonic kidney (HEK) 293 cells (3×10^5^ cells per well in the 12-well plate) were cotransfected with 200 ng of recombinant luciferase reporter plasmid, 50 nM miR-214-3p mimic, and 20 ng of pRL-TK plasmid as an internal control (Promega, Madison, WI). Activities of firefly luciferase (FL) and Renilla luciferase (RL) were measured 24 h after transfection. The relative ratio of the FL/RL was used to indicate the suppression of EZH1 and -2 by miR-214-3p.

### Statistical analysis

The data are presented as the means±s.e.m. In each experiment, all determinations were performed at least in triplicate. Statistical significance between two measurements was determined by the two tailed unpaired Student's *t* test, and among groups, it was determined by one-way ANOVA. A value of *p* < 0.05 was considered to be significant.

## SUPPLEMENTARY MATERIAL


