# Supplementary material for: Targeting EZH1 and EZH2 contributes to the suppression of fibrosis-associated genes by miR-214-3p in cardiac myofibroblasts
Source: Oncotarget. 2016 Nov 3;7(48):78331–42. doi: 10.18632/oncotarget.13048 (PMC5346642; doi:10.18632/oncotarget.13048)
Supplement: Supplementary file 1 [file oncotarget-07-78331-s001.pdf]

Targeting EZH1 and EZH2 contributes to the suppression of fibrosis-associated genes by miR-214-3p in cardiac myofibroblasts

Supplementary Material

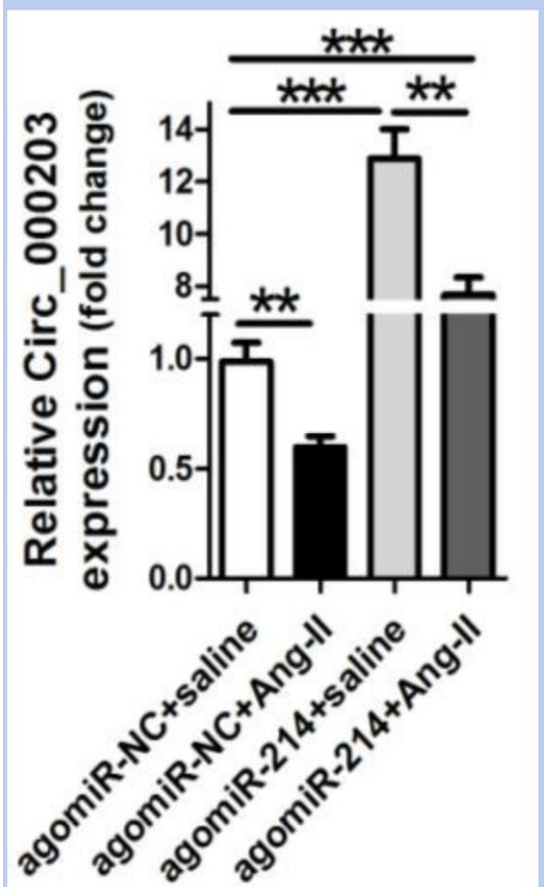

Supp Figure 1 Determination of miR-214-3p in mouse myocardium by RT-qPCR assay. Data are shown as mean ±sem, ***p*<0.01, ****p*<0.001. N=6.

Table 1 Primers used in qRT-PCR assay

Gene	Sequence (5′- 3′)	Product size (bp）
Col1a1	F, CTGGTCCTGTTGGAAGTCGT	201
	R, CAGATGCACCTGTTTCTCCA	
Col3a1	F, CAATGTAAAGAAGTCTCTGAAG	240
	R, CAAACAGGGCCAATGTCCAC	
α-SMA	F, CTGTGCTATGTCGCTCTGGA	192
	R, ATAGGTGGTTTCGTGGATGC	
EZH1	F, GGGACAAAGACATGCAAGCA	198
	R，GTGGTCACAGGGTTGGTAGT	
EZH2	F, TTCCAGCACAAGTCATCCCG	165
	R, TGAAAGTGCCATCCTGATCCA	
PPARγ	F, GTGGGGATGTCTCACAATGC	203
	R, TTTCCTGTCAAGATCGCCCT	
GAPDH	F, CAAGAAGGTGGTGAAGCAGG	200
	R, CCACCCTGTTGCTGTAGCC	

miR-1	RT, GTCGTATCCAGTGCGTGTCGTGGAGT<br>CGGCAATTGCACTGGATACGACATACATACT<br>F, GTCCGCTGGAATGTAAAGAAGTATGTAT<br>R, GTGCGTGTCGTGGAGTC	76
miR-133a	RT, GTCGTATCCAGTGCGTGTCGTGGAGT<br>CGGCAATTGCACTGGATACGACCAGCTGGT<br>F, GTCCGCTTTGGTCCCCTTCAACCAGCTG<br>R, GTGCGTGTCGTGGAGTC	7
miR-133b	RT, GTCGTATCCAGTGCGTGTCGTGGAGT<br>CGGCAATTGCACTGGATACGACTAGCTGGTT<br>F, GTCCGCTTTGGTCCCCTTCAACCAGCTA<br>R, GTGCGTGTCGTGGAGTC	76
miR-16	RT, GTCGTATCCAGTGCGTGTCGTGGAGT<br>CGGCAATTGCACTGGATACGACCGCCAATA<br>F, GTCCGCTAGCAGCACGTAAATATTGGCG<br>R, GTGCGTGTCGTGGAGTC	76
miR-21	RT, GTCGTATCCAGTGCGTGTCGTGGAGT<br>CGGCAATTGCACTGGATACGACTCAACATCA<br>F, GTCCGC TAGCTTATCAGACTGATGTTGA<br>R, GTGCGTGTCGTGGAGTC	76
miR-214-3p	RT, GTCGTATCCAGTGCGTGTCGTGGAGT<br>CGGCAATTGCACTGGATACGACACTGCCTG<br>F, GTCCGCACAGCAGGCACAGACAGGCAGT<br>R, GTGCGTGTCGTGGAGTC	76
miR-29b	RT, GTCGTATCCAGTGCGTGTCGTGGAGT<br>CGGCAATTGCACTGGATACGACAACACTGAT<br>F, GTCCGCTAGCACCATTTGAAATCAGTGTT<br>R, GTGCGTGTCGTGGAGTC	76
U6	RT, GTCGTATCCAGTGCGTGTCGTGGAGT<br>CGGCAATTGCACTGGATACGAC<br>F, GTCCGCGTGCTCGCTTCGGCAGC<br>R, GTGCGTGTCGTGGAGTC	160
